# Prediction of therapy response of breast cancer patients with machine learning based on clinical data and imaging data derived from breast [^18^F]FDG-PET/MRI

**DOI:** 10.1007/s00259-023-06513-9

**Published:** 2023-12-22

**Authors:** Kai Jannusch, Frederic Dietzel, Nils Martin Bruckmann, Janna Morawitz, Matthias Boschheidgen, Peter Minko, Ann-Kathrin Bittner, Svjetlana Mohrmann, Harald H. Quick, Ken Herrmann, Lale Umutlu, Gerald Antoch, Christian Rubbert, Julian Kirchner, Julian Caspers

**Affiliations:** 1 Department of Diagnostic and Interventional Radiology, Medical Faculty and University Hospital Düsseldorf, Heinrich-Heine-University Düsseldorf, Moorenstrasse 5, D-40225 Düsseldorf, Germany; 2 Department Gynecology and Obstetrics, University Hospital Essen, University of Duisburg-Essen, D-45147 Essen, Germany; 3 Department of Gynecology, Medical Faculty and University Hospital Düsseldorf, Heinrich-Heine-University Düsseldorf, D-40225 Düsseldorf, Germany; 4 High-Field and Hybrid MR Imaging, University Hospital Essen, University Duisburg-Essen, D-45147 Essen, Germany; 5 Erwin L. Hahn Institute for Magnetic Resonance Imaging, University Duisburg-Essen, D-45141 Essen, Germany; 6 Department of Nuclear Medicine, University of Duisburg-Essen, and German Cancer Consortium (DKTK)-University Hospital Essen, Essen, Germany; 7 Department of Diagnostic and Interventional Radiology and Neuroradiology, University Hospital Essen, University of Duisburg-Essen, D-45147 Essen, Germany; 8 Center for Integrated Oncology, Aachen Bonn Cologne Düsseldorf (CIO ABCD), Cologne, Germany

**Keywords:** PET/MRI, Breast cancer, Therapy response, Machine learning

## Abstract

**Purpose:**

To evaluate if a machine learning prediction model based on clinical and easily assessable imaging features derived from baseline breast [^18^F]FDG-PET/MRI staging can predict pathologic complete response (pCR) in patients with newly diagnosed breast cancer prior to neoadjuvant system therapy (NAST).

**Methods:**

Altogether 143 women with newly diagnosed breast cancer (54 ± 12 years) were retrospectively enrolled. All women underwent a breast [^18^F]FDG-PET/MRI, a histopathological workup of their breast cancer lesions and evaluation of clinical data. Fifty-six features derived from positron emission tomography (PET), magnetic resonance imaging (MRI), sociodemographic / anthropometric, histopathologic as well as clinical data were generated and used as input for an extreme Gradient Boosting model (XGBoost) to predict pCR. The model was evaluated in a five-fold nested-cross-validation incorporating independent hyper-parameter tuning within the inner loops to reduce the risk of overoptimistic estimations. Diagnostic model-performance was assessed by determining the area under the curve of the receiver operating characteristics curve (ROC-AUC), sensitivity, specificity, positive predictive value (PPV), negative predictive value (NPV), and accuracy. Furthermore, feature importances of the XGBoost model were evaluated to assess which features contributed most to distinguish between pCR and non-pCR.

**Results:**

Nested-cross-validation yielded a mean ROC-AUC of 80.4 ± 6.0% for prediction of pCR. Mean sensitivity, specificity, PPV, and NPV of 54.5 ± 21.3%, 83.6 ± 4.2%, 63.6 ± 8.5%, and 77.6 ± 8.1% could be achieved. Histopathological data were the most important features for classification of the XGBoost model followed by PET, MRI, and sociodemographic/anthropometric features.

**Conclusion:**

The evaluated multi-source XGBoost model shows promising results for reliably predicting pathological complete response in breast cancer patients prior to NAST. However, yielded performance is yet insufficient to be implemented in the clinical decision-making process.

## Introduction

Breast cancer is the most common cancer in women worldwide [1, 2]. Due to improvements in therapy and diagnostics, the 5-year overall survival (OS) rate approaches approximately 90% [3]. Especially for locally advanced- and high-risk breast cancer, accurate pre-therapeutic TNM staging is crucial following the initial diagnosis, as it influences subsequent therapy decisions, despite the determination of histopathological and molecular breast cancer characteristics [4]. Although current guidelines recommend performing chest/abdomen computed tomography (CT) and bone scintigraphy for staging, positron-emission-tomography/magnetic resonance imaging (PET/MRI) has become an increasingly vital diagnostic tool for “one-stop” whole-body staging at leading tumor centers in the last decade [4]. This is mainly due to its ability to combine metabolic imaging with high soft-tissue resolution in a multimodal dataset [5–10].

Current guidelines recommend neoadjuvant system therapy (NAST) for patients with locally advanced- and high-risk breast cancer [11]. The goal of this therapy is to achieve pathologic complete response (pCR) in both, breast cancer primaries and lymph nodes. Pathologic complete response has been shown to be an independent surrogate parameter for overall- and disease-free survival [12, 13]. However, an invasive histopathological assessment after NAST is currently necessary to confirm pCR. Consequently, early and non-invasive identification of breast cancer patients achieving or not achieving pCR during NAST would save a large amount of patients from further surgical treatment and would enable prompt adjustments to potentially toxic and ineffective chemotherapy [7]. Studies have assessed various imaging modalities, such as breast-sonography, mammography, contrast-enhanced MRI, and increasingly PET/computed tomography (CT), for their potential in predicting pCR [14–16]. However, the results of these conventional examinations do not reach the clinically required threshold for reliably predicting pathological complete response in breast cancer patients.

Thus, image-based pCR prediction remains challenging and cannot replace invasive procedures for breast cancer patients in the actual stage of research. However, we hypothesize that advancements in the field of machine learning may offer new possibilities for the non-invasive diagnostic evaluation, as they can detect complex patterns in high-dimensional data. Thus, a lot could be expected from integrating multidimensional data of different modalities. Given the promising results in various medical disciplines, it is unsurprising that machine learning algorithms are increasingly used in image-based data analysis, for example for clinical decision support for axillary lymph node staging in breast cancer patients [17–21]. Unravelling complex patterns within multidimensional datasets, which may be imperceptible to humans, could thus help to predict pCR and potentially elevate diagnostic capabilities in breast cancer patients prior to therapy.

Therefore, this study aims to determine if a machine learning prediction model, utilizing clinical and easily assessable imaging features derived from baseline [^18^F]FDG-PET/MRI staging, can predict pCR in patients with newly diagnosed breast cancer prior to treatment.

## Material and methods

### Patients

The study was approved by the institutional review boards of the University Duisburg-Essen (study number 17-7396-B0) and University Düsseldorf (study number 6040R) and it was performed in accordance with the Declaration of Helsinki [22].

A total of 143 patients were retrospectively included from a trial (register number: DRKS00005410), which included women with newly diagnosed, therapy-naive early breast cancer between March 2018 and December 2021 at the University Düsseldorf and University Duisburg-Essen. Written informed consent was obtained at time of inclusion from all patients. Only breast cancer patients who received and completed a NAST and had immediate subsequent surgery with histologically workup were included. Furthermore, all patients underwent an initial breast-[^18^F]FDG-PET/MRI for staging purposes and met the following, further inclusion criteria: (i) newly diagnosed, therapy-naive T2 or higher T-stage tumor or (ii) newly diagnosed, therapy-naive triple-negative tumor of any size or (iii) newly diagnosed, therapy-naive tumor with a high-risk molecular profile (Ki67 > 14%, G3 or HER2-overexpression).

### [^18^F]FDG-PET/MRI

Patients fasted 6 h prior to the examination to maintain blood glucose levels below 150 mg/dl. The baseline breast-[^18^F]FDG-PET/MRI was performed in head-first prone position on an integrated 3-Tesla PET/MRI system (Biograph mMR, Siemens Healthcare GmbH, Erlangen, Germany) using a dedicated 16-channel radiofrequency (RF) breast coil (Rapid Biomedical, Rimpar, Germany) [23]. PET and MRI data of both breasts were acquired simultaneously with a 20-min acquisition time per bed position.

The full diagnostic breast-MRI protocol comprised the following sequences:

- A transversal T2-weighted (T2w) Turbo-spin Echo (TSE) fat-saturated sequence with a slice thickness of 7 mm (TE 97 ms; TR 2840 ms; FOV 400 mm; phase FOV 75%; acquisition matrix 256 × 192, in-plane resolution 1.6 × 1.6 mm.^2^)
- A transversal diffusion-weighted echo-planar imaging (EPI) sequence with a slice thickness of 5.0 mm (TR 8000 ms; TE 81 ms; *b*-values: 0, 400, and 800 s/mm^2^, matrix size 192 × 156; FOV 420 mm, phase FOV, 81.3%; GRAPPA, acceleration factor 2; in-plane resolution 2.2 × 2.2 mm.^2^)
- Six repetitions of a transversal 3-dimensional fast low-angle shot (FLASH) T1w sequence with a slice thickness of 7 mm (TE 3.62 ms; TR 185 ms; FOV 400 mm; phase FOV 75%; acquisition matrix 320 × 240, in-plane resolution 1.3 × 1.3 mm^2^) for dynamic contrast-enhanced imaging. A dose of 0.2 mmol/kg body weight gadoterate meglumine (Guerbet, Dotarem®, Sulzbach, Germany) was injected intravenously after the first FLASH sequence with a flow of 2 mL/s using an automated injector (Spectris Solaris, MR Injection System; Medrad, Pittsburg, PA). Subsequent automated image subtraction was performed.

For attenuation correction (AC) of the patient tissue a Dixon VIBE MR sequence was used [24]. MR images of the Dixon-VIBE sequence were automatically segmented into four tissue classes (background air, lung, fat, and soft tissue) with pre-defined linear attenuation coefficients. The resulting AC-map was completed with a bone atlas and truncation correction [25, 26]. For the RF breast coil AC, a registered CT-based AC-map was implemented on the PET/MR system [23]. PET image reconstruction was performed by using an iterative ordered subset expectation maximization algorithm with 3 iterations and 21 subsets, a Gaussian filter with 4-mm full width at half maximum, and a 256 × 256 image matrix. The resulting PET images had a matrix size of 344 × 344 × 127 and a resolution of 2.09 mm × 2.09 mm × 2.03 mm per bed position.

### Image analysis

All breast-[^18^F]FDG-PET/MRI datasets were analyzed using OsiriX (version 9.0.2; Pixmeo SARL, Bernex, Switzerland) in random order. Two readers with more than two and ten years of experience in breast- and hybrid imaging performed data evaluation in consensus.

**Fig. 1:**
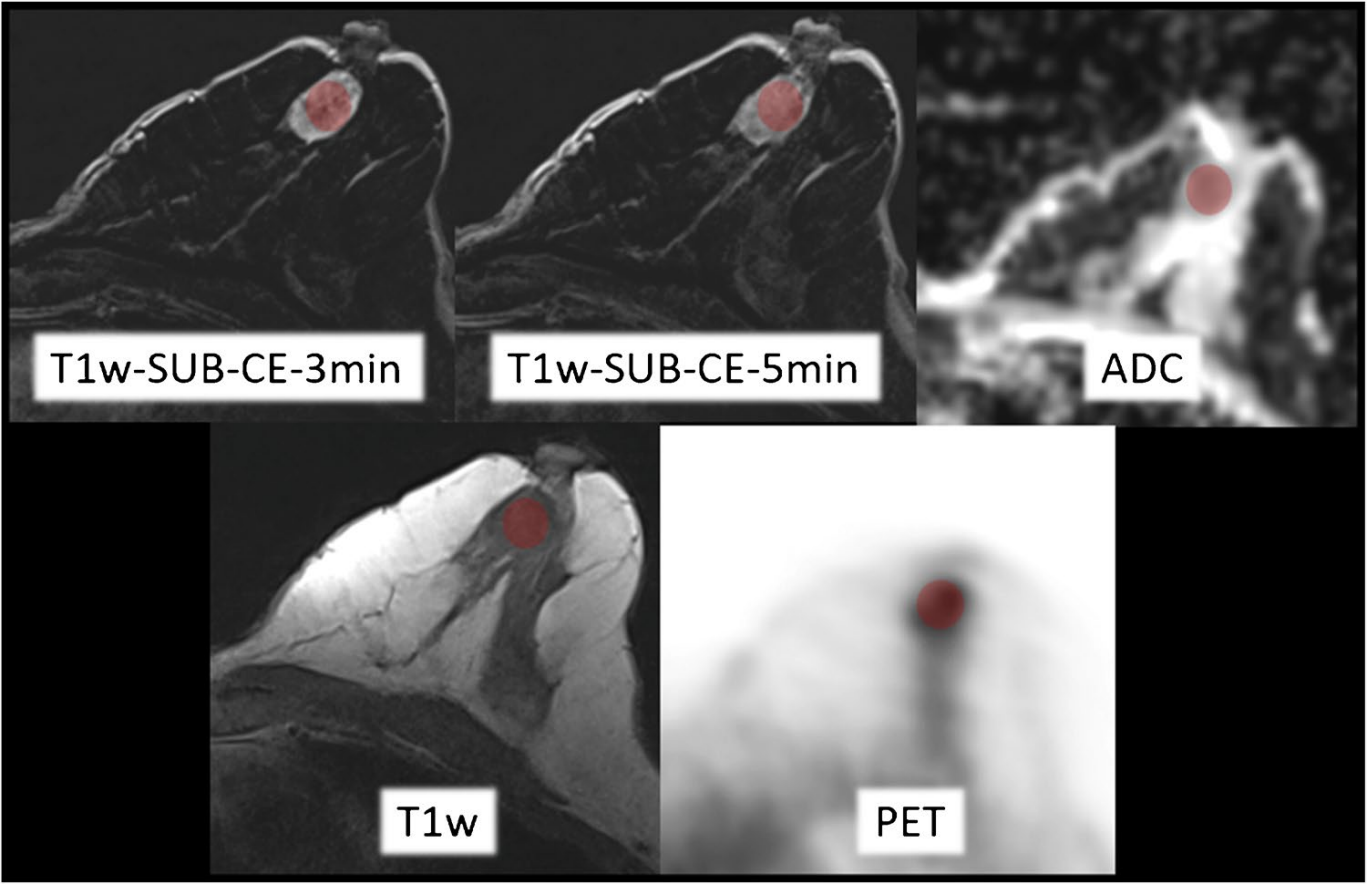
Example of the measuring procedure of a malign breast cancer lesion at different sequences of breast-[^18^F]FDG-PET/MRI dataset. The red circle represents the volume of interests (VOI)

Tumor size (mm) was measured on T1w post-contrast images in three dimensions. Additionally, tumor imaging features, detailed below, were measured using a tumor size adapted spherical volume of interest (VOI) that captured the breast cancer lesion as defined on the T1w post-contrast images. The predefined VOI was copied to each sequence of the individual PET/MRI imaging dataset to match the identical plane and position. When movement during the examination was noted, or when the VOI did not optimally align with the lesion due to distortion artifacts, the VOI was manually reshaped. For an example, see Fig. 1.

### Patient demographics/characteristics and histopathological parameters

In addition to imaging data, specific patient history, clinical data, and histopathologic data of the primary tumor were collected for each patient (see Table 1). Adapting the World Health Organization classification after ultrasound guided biopsy, tumor grading (G1-G3), tumor type, and tumor biology, including estrogen (ER) and progesterone receptor (PR) as well as human epidermal growth factor receptor 2 (HER2) status, and Ki67 (proliferation marker), were recorded.

**Table 1:** Overview of patient characteristics used as features for the machine learning approach

Patient demographic/characteristic	Value
Number of patients	*n* = 143
Age (years)	
Mean ± SD	54 ± 12
Height (cm)	
Mean ± SD	167 ± 7
Weight (kg)	
Mean ± SD	71 ± 14
Body surface (cm^2^)	
Mean ± SD	17918 ± 1763
State of menopause	
pre-menopause	*n* = 66/143 (46%)
peri-menopause	*n* = 14/143 (10%)
post-menopause	*n* = 63/143 (44%)
Familiar breast cancer risk	*n* = 34/143 (24%)
TNM staging (tumor)	
T1	*n* = 48/143 (33%)
T2	*n* = 83/143 (58%)
T3	*n* = 5/143 (4%)
T4	*n* = 7/143 (5%)
TNM staging (nodus)	
N1	*n* = 37/143 (26%)
N2	*n* = 2/143 (1%)
N3	*n* = 14/143 (10%)
Histopathological status	
ER +	*n* = 96/143 (67%)
PR +	*n* = 93/143 (65%)
HER2 +	*n* = 39/143 (27%)
KI67 > 14%	*n* = 137/143 (96%)
G1	*n* = 1/143 (1%)
G2	*n* = 74/143 (52%)
G3	*n* = 68/143 (47%)
Molecular subtype	
Luminal A	*n* = 1/143 (1%)
Luminal B	*n* = 107/143 (75%)
HER2 +	*n* = 3/143 (2%)
TNBC	*n* = 32/143 (22%)
Pathological complete response to NAST	
Yes (Sinn 4)	*n* = 51/143 (35.7%)
No (Sinn 0–3)	*n* = 92/143 (64.3%)

### Reference standard

Histopathological workup of surgical resected breast cancer specimens after NAST served as a reference standard to distinguish between pCR and non-complete pathological response (non-pCR). Regression criteria defined by Sinn et al. (1994) were used to assess therapy response [27]. Sinn regression grades were dichotomized into “tumor detectable” (Sinn regression grades 0 to 3) vs. “tumor not detectable” (Sinn regression grade 4) to gain a binary variable for classification. The dichotomized Sinn regression grade served as the outcome variable for machine learning model development.

### Feature definition

A total of 56 features were generated to be used in a machine learning model.

- Histopathological features consisting of the following: estrogen receptor measured in % as well as binary feature (yes/no); progesterone receptor measured in % as well as binary feature (yes/no); Ki67 measured in % and implemented as one-hot-encoded using three different thresholds (> 14%, > 20%, > 30%); HER2 expression divided into no, poor, moderate, and strong expression; HER2 positivity (HER2 +) as binary feature (yes/no) according to the actual guideline definitions [4]; histological grade divided into G1, G2, and G3; molecular subtype divided into Luminal-A-like, Luminal-B-like, triple negative breast cancer (TNBC), and HER2 + .
- PET features consisting of the following: visual PET positivity (clearly delineated [^18^F]FDG enhancement of breast cancer lesion compared to surrounding breast parenchyma) implemented as binary feature (yes/no); SUVmax and SUVmean values of the breast cancer lesion; ratios of SUVmax/SUVmean of the breast cancer lesion to SUVmax/SUVmean of blood pool measured in the proximal descending aorta, respectively; SUVmax/SUVmean of the lesion to SUVmax/SUVmean of the liver measured in the right hepatic lobe; SUVmax of the lesion to SUVmax of breast parenchyma measured in the same quadrant of the opposite site (SQOS); SUVmax of the lesion to SUVmax of breast parenchyma measured in the opposite quadrant at the same site (OQSS).
- MRI features consisting of the following: length of the breast cancer lesion measured at 3 min T1w post-contrast sequence; height of the breast cancer lesion measured at 3 min T1w post-contrast sequence; width of the breast cancer lesion measured at 3 min T1w post-contrast sequence; volume of the breast cancer lesion measured at 3 min T1w post-contrast sequence; relative contrast enhancement of breast cancer lesions at 3 min and 5 min compared to non-contrast non-fat saturated T1w-sequence; ADCmean value of the breast cancer lesion; ratios of the non-fat saturated, non-contrast T1w value of the breast cancer lesion to the pectoral muscle on the same side; ADCmean of the breast cancer lesion to ADCmean of breast parenchyma measured in the same quadrant of the opposite site; visual assessment of fibroglandular tissue divided into almost entirely fatty, scattered areas of fibroglandular density, heterogeneously dense and extreme dense; visual assessment of breast parenchyma enhancement divided into minimal, poor, moderate, and strong.
- Sociodemographic and anthropometric features consisting of the following: age; weight; height; body mass index (BMI); body surface according to Dubois [28]; binary variable of familiar risk for breast cancer (yes/no); state of menopause divided into pre-menopause, peri-menopause, post-menopause.
- “Other” features consisting of the following: site of breast cancer lesion; nodal involvement (N-status); T-status; UICC state broadly divided into 1, 2, 3, and 4; binary breast cancer NAST features consisting of the following: cyclophosphamid (yes/no); carboplatin (yes/no); epirubicin (yes/no); etoposid (yes/no); docetaxel (yes/no); paclitaxel (yes/no); tamoxifen (yes/no); GNRH (yes/no); aromatase inhibitors (yes/no); monoclonal antibodies (yes/no); a grouped feature of breast cancer NAST combinations divided into nine present therapeutic groups (group 1: paclitaxel, epirubicin and cyclophosphamid; group 2: paclitaxel, epirubicin, cyclophosphamid, carboplatin, and monoclonal antibodies; group 3: paclitaxel, etoposid, cyclophosphamide, and monoclonal antibodies; group 4: paclitaxel, carboplatin; group 5: cyclophosphamid, docetaxel; group 6: epirubicin, cyclophosphamid; group 7: paclitaxel, epirubicin, carboplatin, and monoclonal antibodies; group 8: paclitaxel and monoclonal antibodies; group 9: paclitaxel, cyclophosphamid, carboplatin, and monoclonal antibodies).

**Fig. 2:**
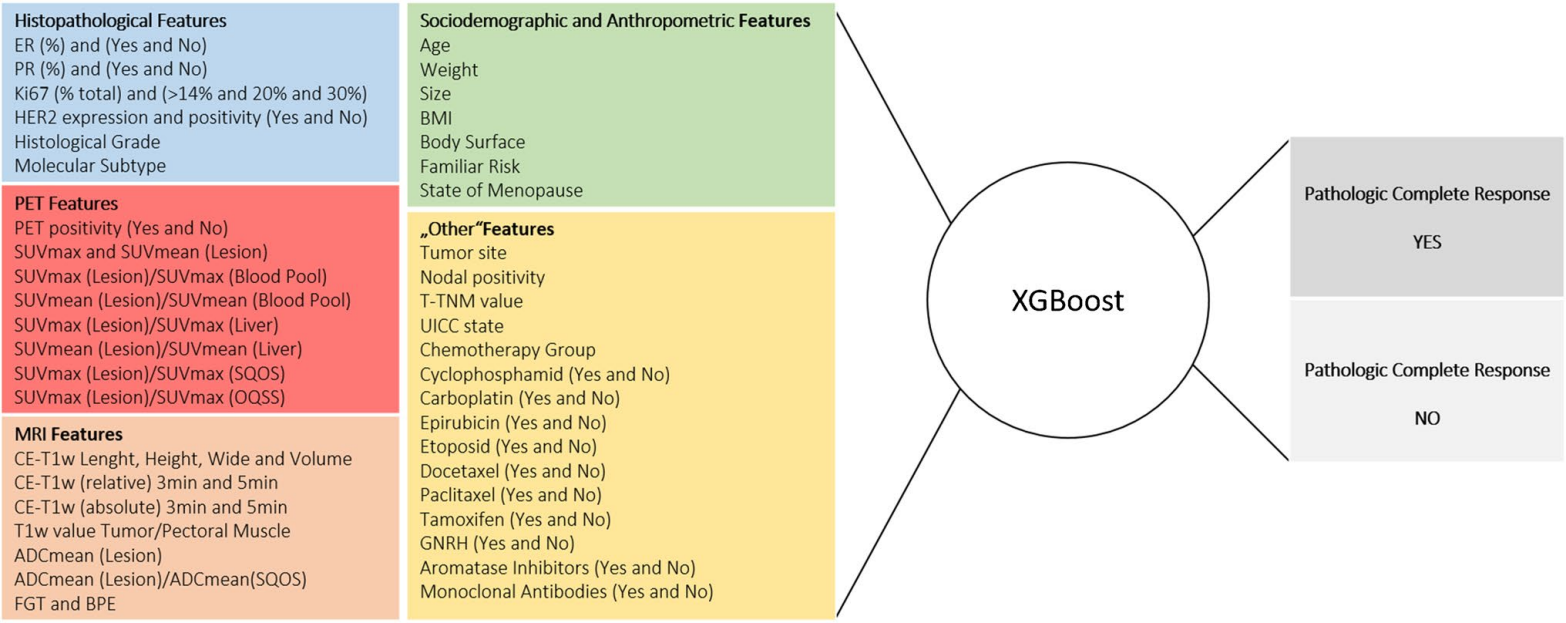
Imaging data derived from PET/MRI used for further AI evaluation. The features are divided in histopathological features (blue), PET features (red), MRI features (beige), sociodemographic/anthropometric features (green), and “other” features (yellow). Outcome variables of XGBoost are divided in pathologic complete response yes (darker grey) and pathologic complete response no (lighter grey)

An overview of the implemented features is given in Fig. 2.

### Model development

An eXtreme Gradient Boosting (XGBoost) model was deployed to predict treatment response after NAST. XGBoost is a tree-based gradient boosting algorithm, i.e. a machine learning technique that builds an ensemble of weak decision tree models and combines them to create a stronger model. One of the main advantages of XGBoost is its flexibility in handling various data types and formats without the need for elaborate feature engineering or feature reduction techniques. Another advantage is its high performance on structured data problems, where XGBoost often outperforms other algorithms with regard to predictive power. The imaging and non-imaging features of each breast cancer patient, as specified above, served as input to the model. The dichotomized modified Sinn regression grade (“tumor detectable” vs. “tumor not detectable”) was set as output variable for predictive modeling. A decision threshold determines the probability boundary for classifying outcomes; for instance, with a threshold of 0.5 in binary classification, probabilities at or above 0.5 are classified as the positive class, and those below as the negative class. The XGBoost model was trained, optimized, and evaluated using a nested cross-validation performed on the whole dataset. This approach allows to estimate the generalization performance on unseen data with optimized hyperparameters while avoiding data leakage. The process involves two rounds of cross-validation: An outer loop, where the performance of the model is evaluated across the hold-out datasets of a k-fold cross-validation, and an inner loop, where the hyperparameters of the models are independently tuned in each iteration of the outer loop. For the current study, we used a five-fold cross-validation for the outer loop with five random 80:20 splits of the whole dataset stratified for the outcome variable. Each of the “train” splits of the outer loop runs is then subjected to another five-fold stratified cross-validation, the so-called inner loops, to identify the optimal hyperparameters for the XGBoost model using a random search with 100 iterations optimizing for the largest area under the curve of the receiver operating characteristics (ROC-AUC) [29]. Hyperparameters considered for optimization comprised the number and the maximum depth of trees in XGBoost, the learning rate, gamma (i.e., the minimum loss reduction for a partition in a tree), the subsample ratio for instances and features (“colsample_bytree”), and the number of boosting rounds for evaluation of early stopping. For each split of the outer loops, the model was refit on the whole training data of that (outer loop) split using the optimized hyperparameters from the respective inner loop and then tested on the hold-out data of the outer loop. The mean predictive performance across the five outer loop splits for prediction of pCR was assessed by determining mean ROC-AUC, sensitivity, specificity, positive predictive value (PPV), and negative predictive value (NPV) and accuracy. Additionally to the default decision threshold of the XGBosst model at 0.5, more conservative (threshold at 0.7) or lenient (threshold at 0.3) decision thresholds were tested and the aforementioned metrics were evaluated for these cut-off values. Despite total data evaluation a further subgroup analysis focusing on HER2+ patients was conducted by employing another XGBoost model on this subsample using an equivalent model deployment and evaluation as for the total sample.

Model development and evaluation was conducted using Python v3.10.4 with the scikit-learn library v1.1.2 and using the XGBoost implementation for Python v1.7.2 (https://github.com/dmlc/xgboost/).

### Feature importances

To evaluate which features contributed most to the classification of pCR and non-pCR, a built-in function of XGBoost was used to analyze feature-importance and averaged across the outer splits of nested cross-validation. It is based on the “gain” of each feature, i.e. the average gain in classification accuracy of the tree’s splits which use the feature for each tree in the model. A higher gain for a feature implies that it is more important for generating a valid prediction.

## Results

### Patient demographics/characteristics

Fifty-one of 143 (35.7%) patients achieved pCR according to the reference standard and 92/143 (64.3%) patients were designated non-pCR. A detailed overview of the demographics and patient characteristics is provided in Table 1.

### XGBoost model performance

Nested-cross-validation employing the XGBoost model on total data yielded a mean ROC-AUC of 80.4 ± 6.0% (range: 71.7 to 86.3%) for the prediction of pCR across the outer loops. With the default decision threshold at 0.5, a mean sensitivity (recall) of 54.5 ± 21.3% (range: 30.0 to 72.7%), a mean specificity of 83.6 ± 4.2% (range: 77.7 to 89.5%), a mean PPV (precision) of 63.6 ± 8.5% (range: 55.5 to 72.7%), a mean NPV of 77.6 ± 8.1% (range: 70.8 to 88.9%), and a mean accuracy of 73.3 ± 6.4% (range: 67.8 to 82.8%) could be achieved. After adapting the decision cut-off to 0.7, mean sensitivity drops to 21.4 ± 17.0% (range: 0.0 to 100.0%) while mean specificity increases to 98.9 ± 2.1% (range: 94.7 to 100.0%), mean PPV increases to 76.7 ± 38.9% (range: 0.0 to 100.0%), mean NPV slightly decreases to 69.8 ± 4.5% (range: 65.5 to 78.2%), and mean accuracy is quite stable with 71.3 ± 4.7% (range: 65.5–79.3%). Lowering the decision cut-off to 0.3 yielded an increased mean sensitivity of 90.3 ± 8.6% (range: 81.8 to 100.0%), a decreased mean specificity of 51.3 ± 26.9% (range: 0.0 to 78.9%), a slightly decreased mean PPV of 53.8 ± 11.1% (range: 34.5 to 69.2%), and an increased mean NPV of 90.7 ± 36.8% (range: 0.0 to 100.0%). The mean accuracy was slightly decreased to 65.1 ± 16.2% (range: 34.5–82.8%). For a graphical overview, see Table 2.

**Table 2:** Performance of the XGBoost model for predicting pathological complete response (pCR) vs. non-pCR on total data. Performance metrics were calculated across the outer loops of a fivefold nested cross-validation. Mean, standard deviation, and ranges are reported for ROC-AUC, sensitivity, specificity, positive predictive value (PPV; precision); negative predictive value (NPV) and accuracy. Decision cut-off values of 0.3, 0.5, and 0.7 were evaluated

Performance criteria	XGBoost model		XGBoost model		XGBoost model	
	cut-off 0.5		cut-off 0.3		cut-off 0.7	
	mean ± SD (range)		mean ± SD (range)		mean ± SD (range)	
ROC-AUC	80.4 ± 6.0%	(71.7–86.3%)	80.4 ± 6.0%	(71.7–86.3%)	80.4 ± 6.0%	(71.7–86.3%)
Sensitivity	54.5 ± 21.3%	(30.0–72.7%)	90.3 ± 8.6%	(81.8–100.0%)	21.4 ± 17.0%	(0.0–100.0%)
Specificity	83.6 ± 4.2%	(77.7–89.5%)	51.3 ± 26.9%	(0.0–78.9%)	98.9 ± 2.1%	(94.7–100.0%)
Positive predictive value (PPV)	63.6 ± 8.5%	(55.5–72.7%)	53.8 ± 11.1%	(34.5–69.2%)	76.7 ± 38.9%	(0.0–100.0%)
Negative predictive value (NPV)	77.6 ± 8.1%	(70.8–88.9%)	90.7 ± 36.8%	(0.0–100.0%)	69.8 ± 4.5%	(65.5–78.2%)
Accuracy	73.3 ± 6.4%	(67.8–82.8%)	65.1 ± 16.2%	(34.5–82.8%)	71.3 ± 4.7%	(65.5–79.3%)

A separate subgroup analysis focusing on HER2+  patients employing the XGBoost model yielded a mean ROC-AUC of 85.6 ± 12.2% (range: 61.5 to 93.8%) for the prediction of pCR across the outer loops. A mean sensitivity of 35.0 ± 27.9% (range: 0.0 to 75.0%), a mean specificity of 95.4% ± 9.2% (range: 76.9 to 100.0%), a mean PPV of 65.0 ± 43.6% (range: 0.0 to 100.0%), a mean NPV of 79.9% ± 8.4% (range: 71.4 to 92.9%), and a mean accuracy of 78.8 ± 11.8% (range: 61.1–94.1%) could be achieved.

### Feature importance

**Fig. 3:**
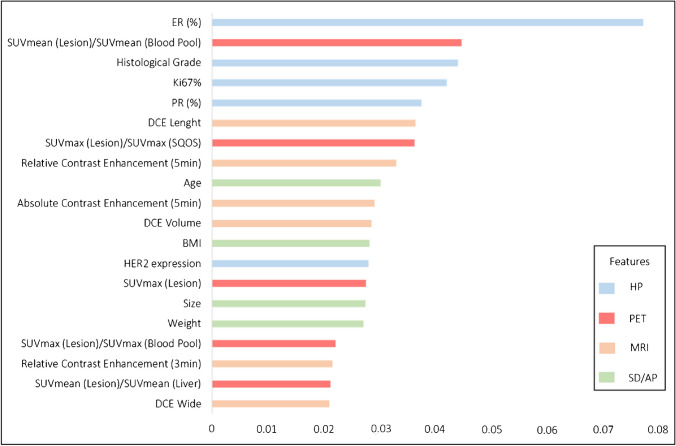
The twenty most important features to the predictive performance of the XGBoost model across the outer loops of the nested cross-validation. The features are divided in histopathological (HP, blue), PET (red), MRI (beige), and sociodemographic/anthropometric (SD/AP, green) features. X-axis visualizes the rate of feature importance

The rank of the 20 most important features contributing to the performance of the XGBoost model across the nested cross validation are visualized in Fig. 3.

With regard to the distinct data sources of the features, histopathological features (ER+ measured in %; histological grade ranging from G1 to G3; Ki67 measured in %; PR+ measured in %) contributed to four of the five most important features. The most important image based feature was the ratio of SUVmean of the lesion to SUVmean of the proximal descending aorta (blood pool) and contributed as second most important feature of the XGBoost model. PET and MRI features contributed with a nearly equal feature importance and consisted of the following PET features: (i) the ratio of SUVmax of the lesion to SUVmax of the breast parenchyma measured in the same quadrant of the opposite site (SQOS) and (ii) SUVmax of the lesion (iii) the ratio of SUVmax of the lesion to SUVmax of the proximal descending aorta (blood pool) (iv) the ratio of SUVmean of the lesion to SUVmean of the liver and following MRI features: (i) the tumor length (ii) relative contrast enhancement of the tumor at 5 min T1w post-contrast sequence (iii) absolute contrast enhancement of the tumor at 5 min T1w post-contrast sequence (iv) the tumor volume (v) relative contrast enhancement of the tumor at 3 min T1w post-contrast sequence (vi) the tumor width. Overall, features of the sociodemographic and anthropometric subgroup contributed less than histopathologic and nearly equal to several PET and MRI imaging features, whereas age, weight, size, and BMI were the most important sociodemographic and anthropometric factors.

## Discussion

At locally advanced or high-risk breast cancer, NAST is recommended as the therapy of choice in current breast cancer guidelines as part of a multimodal therapeutic approach [11]. The goal of this neo-adjuvant therapy regimen is the achievement of a pCR, which is considered an independent surrogate parameter for overall- and disease-free survival [12, 13]. However, the major problem during such intense therapies are adverse effects and complications increasing with patient’s age, accomplished by naturally decreasing resilience [30]. Although several conventional studies examined histopathological data and different imaging modalities for their potential in predicting pCR [14–16, 31, 32], by now, only histopathological workup of resected breast cancer tissue after NAST provides adequate sensitivity.

For that purpose, this study presents and evaluates an XGBoost-based machine learning model for the prediction of pCR based on clinical, histopathologic, and easily assessable multimodal imaging features derived from baseline breast [^18^F]FDG-PET/MRI prior to NAST in breast cancer patients to predict pCR on an individual basis, which may contribute to an improved patient-centered therapy.

According to the presented data, three important observations can be derived: First, histopathological data including receptor state (ER and PR), histological grade, and proliferation index (Ki67) were the most important features for the predictive performance of the XGBoost model. This is consistent with actual clinical decision making in routine clinical practice. Here, the individual histopathological profile of the breast cancer has a decisive influence on the choice of NAST. Thus, for example, endocrine target therapies are crucial in hormone receptor positive breast cancer [4].

Second, metabolic (PET) features show a high feature importance in the XGBoost model and the ratio of SUVmean of the lesion to SUVmean of the proximal descending aorta (blood pool) contributed as second most important feature of the model, outperforming all MRI features and shows similar importance to several histopathologic features. This could be explained by the representation of tumor metabolism, which is also influenced by tumor biology and thus allows indirect conclusions about the effect of therapy [33]. Previous studies have shown correlations between metabolism (SUV) and tumor biology in conventional statistical analysis, further bolstering this understanding. For instance, Catalano et al. (2017) identified an inverse correlation between the SUVmax of the BC lesion and both estrogen receptor and progesterone receptor expression, a finding also supported by other research [34, 35]. Additionally, correlations of SUV values with Ki67 and tumor grading hint at potential ties to tumor aggressiveness [35].

Third, the XGBoost model achieved a mean ROC-AUC of about 80% across a nested cross-validation, indicating a good predictive performance of the approach to unseen data. These results are comparable to a deep-learning based approach published by Choi et al. (2020), yielding a ROC-AUC of 80% by implementing a deep learning model based on PET data from PET/CT and breast MRI data [36]. Deviating from our study, Choi and colleagues used imaging data collected before and after NAC and did not include other patient data. Our approach achieves comparable predictive performance only with data collected before NAST. This is particularly noteworthy because, in the absence of external validation data, our study used a nested cross-validation approach, which is a rather conservative method in regard to estimating the generalizability of a model to given data due to reduced risk of overestimation/overfitting of a machine learning model [37–39].

It should be mentioned that the XGBoost model (cut-off 0.5) yielded a relatively large spread of sensitivity (30–72.7%), specificity (77.7–89.5%), PPV (55.5–72.7%), and NPV (70.8–88.9%) across the random splits of the nested cross-validation, most likely owed to a rather high subtype heterogeneity of the included sample as well the heterogeneity of performed NAST regimes. Although this study included a relatively large collection of high-risk breast cancer patients imaged with [^18^F]FDG-PET/MRI, a much larger patient cohort might homogenize the results and also improve the model performance in some cases. Nonetheless, there is the risk that variance and heterogeneity of the data could also increase after including much more patients and data. This could negatively affect the model performance and results and must be considered even if larger cohorts and datasets are used. Nevertheless, testing such predictive models in large samples with a wide range of variance will be important to better estimate the generalizability of a model’s performance in real-life scenarios [40]. Furthermore, according to previous studies, repetitive imaging during NAST seems to be a promising approach and is expected to increase the predictive performance [41, 42]. For example, Syed et al. (2023) achieved an AUC of 95% using longitudinal MRI features, tumor characteristics, and patient demographics in their XGBoost model [42]. Since our evaluation showed a high rank of the PET component with respect to the feature importance, it can be assumed that the predictive power can be further increased with additional longitudinal parameters. Furthermore, sensitivity might also benefit from longitudinal imaging, as Syed et al. (2023) demonstrated with a sensitivity of 93% in their study [42].

Predicting NAST non-responders in breast cancer patients may also be of great clinical importance for therapeutic decision making. Early identification of a non-responding patient could lead to early alternative treatment planning in case of severe side effects. In this scenario, high negative predictive values and high specificities are more desirable than high sensitivities, which is the case in our model. However, the mean NPVs (77.7%) and specificities (83.6%) obtained are not yet high enough to justify the use of such an approach as a single marker for clinical decision making, but can be used to guide decisions in case of doubt. Additionally, with regard to the presented data on differing decision cut-offs, threshold modifications could be helpful adjust a model in a direction to more appropriately answer specific clinical questions. Thus, for example, lowering the cut-off to 0.3 increased the NPV to 90.7%.

Future research should focus on reliably predicting pCR and non-PCR in breast cancer patients by leveraging the huge amount of multimodal data already available. With respect to potentially increasing variance and heterogeneity of data, increasing the number of patients could be one possibility for model improvement. A reduction of implemented features would probably not increase model performance in the used gradient boosting approach that is not very prone to feature redundancy and already entails an implicit feature selection. Based on the current literature and the presented data, integrating (even short-term) follow-up scans might add substantially important information for the decision task, which could probably elevate model performance metrics to another level. Furthermore, integrating information from axillary lymph node involvement/pCR would be important to get a more integral view of therapy response after NAST [17]. Following this could significantly improve individualized and patient-centered decision making in favor and against a NAST in breast cancer patients.

There are some limitations to this study. Although it has to be noted that in light of the limited availability of breast [^18^F]FDG-PET/MRI datasets, this study includes a relatively large number of patients, including a larger number of patients might be beneficial. A second limitation is the present inhomogeneity, especially with regard to the histopathological characteristics or NAST therapy regimes. Although the presented subgroup analysis of HER2+ patients not reach the increase of model performance Umutlu et al. (2022) mentioned in their analysis, it could be a hint that sample heterogeneity maybe one of the relevant factors responsible for only moderate predictive performances in the main analysis. Nevertheless, it has to be mentioned that the number of patients in this subgroup analysis is rather low, which may affect reliability of the observed results. Nonetheless, the risk of inhomogeneity of course is consistent with the clinical reality of breast cancer patients, but underlines the need for a large number of patients to include in a machine learning–based approach so that as many combinations as possible can be learned by the model. Furthermore, analysis of feature importance should be interpreted with respective caution in regard to possible dilution effects in case of collinearity of included features. However, such dilution effect due to collinearity is mainly a problem of parallel ensemble learning classifiers (e.g., random forest) and can be largely avoided in the gradient boosting models like the one used for the current study.

## Conclusion

In conclusion, the evaluated multimodal machine learning model shows promising results for predicting pathological complete response in breast cancer patients prior to NAST, but yielded results are currently insufficient to be implemented in the clinical decision-making process.

Future studies with larger patient cohorts and longitudinal breast [^18^F]FDG-PET/MRI data during NAST may be helpful to develop clinical valid models.

## Data Availability

The datasets used and/or analyzed during the current study are available from the corresponding author on reasonable request.
